# Evaluation of iron transport from ferrous glycinate liposomes using Caco-2 cell model

**DOI:** 10.4314/ahs.v17i3.37

**Published:** 2017-09

**Authors:** Ding Baomiao, Yi Xiangzhou, Li Li, Yang Hualin

**Affiliations:** College of Life Science, Yangtze University, Jingmi Road 266, Jingzhou Hubei 434025, China

**Keywords:** Ferrous glycinate liposomes, iron transport, phytic acid, particle size

## Abstract

**Background:**

Iron fortification of foods is currently a strategy employed to fight iron deficiency in countries. Liposomes were assumed to be a potential carrier of iron supplements.

**Objective:**

The objective of this study was to investigate the iron transport from ferrous glycinate liposomes, and to estimate the effects of liposomal carriers, phytic acid, zinc and particle size on iron transport using Caco-2 cell models.

**Methods:**

Caco-2 cells were cultured and seeded in DMEM medium. Minimum essential medium was added to the basolateral side. Iron liposome suspensions were added to the apical side of the transwell.

**Results:**

The iron transport from ferrous glycinate liposomes was significantly higher than that from ferrous glycinate. In the presence of phytic acid or zinc ion, iron transport from ferrous glycinate liposomes and ferrous glycinate was evidently inhibited, and iron transport decreased with increasing phytic acid concentration. Iron transport was decreased with increase of particle size increasing of ferrous glycinate liposome.

**Conclusion:**

Liposomes could behave as more than a simple carrier, and iron transport from liposomes could be implemented via a mechanism different from the regulated non-heme iron pathway.

## Introduction

Iron deficiency is a common nutritional disorder problem worldwide^1^. Insufficient dietary intake and low iron bioavailability in foods are usually the primary contributing causes of iron deficiency^2^,^3^. Iron fortification of foods is currently a strategy employed to fight iron deficiency in countries^4^. Iron supplements utilized in food fortification should be readily bioavailable, stable and safe^5^. Liposomes are potential carriers^6^,^7^, and the delivery systems have been used to increase iron absorption in certain food matrices such as dairy products^8^.

Most of traditional iron supplements could injure the mucosa of the gastrointestinal tract, and high iron intake could also lead to iron overload in some human being, which could result in cell toxicity and side effects such as respiratory morbidities, nausea, abdominal discomfort, constipation, and an increased risk of infection. Compared with common iron supplements, iron liposomes can obviously increase the iron levels and haemoglobin concentrations in serum so as to alleviate the anemia^9^,^10^. Liposomal iron did not aggravate the oxidative stress at the same time of increasing iron levels in serum. Moreover, iron liposomes are microvesicles and mostly nanosized particles, they also have physical stability and gradual release properties. Furthermore, they have no toxicity and minimal side effects to the body than unencapsulated iron supplements^7^,^8^.

The absorption of non-heme iron, such as ferrous sulphate, could be restrained by several factors. Phytic acid in diets based on cereals and legumes has been shown to inhibit iron absorption in humans and in cell culture models^11^. It has also been proved that some divalent metal ions (such as Zn^2+^) decreased iron uptake^12^. The core material bioavailability could be improved by liposomes^8^, and the delivery efficiencies of liposomes to core materials are significantly influenced by liposomal physico-chemical properties, such as particle size^13^. However, the effects of these factors on the regulation of iron transport by liposomes are still obscure.

The Caco-2 cells, a human adenocarcinoma cell line, shows promise as a rapid and low-cost model to predict iron absorption from foods and iron fortificants^14^,^15^. The model system has been applied numerously to estimate relative iron bioavailability from varieties of staple food crops, commercial food products, and meals or specific food, etc^15^,^16^. To further assess the regulation of liposomes, iron transport experiments with ferrous glycinate liposomes were performed using Caco-2 cell model.

Ferrous glycinate as an iron chelate has been microencapsulated using liposomes in previous work, and the pH stability of the iron supplement obviously increased^17^, ^18^. The objective of the present study was to evaluate the regulation of iron transport by liposomes using Caco-2 cell model, and to provide information on the effects of known inhibitors of iron transport from ferrous glycinate liposomes (i.e. phytic acid and zinc), and to determine the effects of liposome particle size. Documentation of these results will be valuable to understand the regulation of liposomes on iron transport and to predict the iron availability of ferrous glycinate liposomes.

## Material and methods

### Materials

Egg phosphatidylcholine (EPC), cholesterol, Tween 80 and diethyl ether were purchased from Sinopharm Chemical Reagent Co., Ltd (Shanghai, China). Minimum essential medium (MEM medium), D-Hanks buffer, phytic acid and ZnCl_2_ were obtained from Sigma-Aldrich Co., LLC (Shanghai, China). All chemicals were of reagent grade and used without further purification. Ferrous glycinate was synthesized according to CN Patent ZL200410065260.3.

### Preparation of ferrous glycinate liposomes

Ferrous glycinate liposomes were prepared by reverse phase evaporation (REV) method^19^. Briefly, the lipid mixture, containing EPC (200 mg) and cholesterol (20 mg), was dissolved in 10 mL diethyl ether (organic phase). Ferrous glycinate (40 mg) was dissolved in 3 mL aqueous solution (phosphate buffer solution, PBS, 0.05 mol/L, pH6.8). The aqueous phase was added to the organic phase, and then a homogeneous w/o emulsion was obtained by ultrasonication with a probe sonicator (VCX400, Sonics & Material, USA) with a sequence of 1 s on and 1 s off (sonication power 300 W) in an ice bath for 5 min. The w/o emulsion in a round bottom flask was evaporated using a rotary evaporator under reduced pressure at 40°C, and a gel was formed. Upon further rotary evaporation, the gel was broken, and then 10 mL PBS buffer (0.05 mol/L, pH6.8) containing 100 mg Tween 80 was added with gentle vortexing and the sample was sequentially evaporated for 30 min at 40°C. The residual diethyl ether was evacuated by nitrogen gas.

### Procedures to purify ferrous glycinate liposomes and to control liposomal particle sizes

The purification and particle size control of ferrous glycinate liposomes was processed simultaneously. Firstly, liposomes were separated according to the particle size by Sephadex G-100 column (20 cm × 1 cm id), and the samples were eluted using 0.9% (w/v) NaCl solution, and the eluate was collected by test tubes per minute. Secondly, liposomes were extruded to adjust particle sizes using an ultrafiltration cell (Amicon stirred cell 8010, Millipore, USA) with a polycarbonate membrane (1.0, 0.6, 0.4, 0.2, and 0.1 µm pores). Samples were extruded through a membrane using N_2_ gas, and extruded solution was collected. The obtained solution could be further extruded to reduce the particle size using a membrane with next smaller pore size. At last, the first step was repeated to eliminate free iron. Particle size and size distribution were measured by dynamic light scattering with a ZEN3600 Zetasizer nano instrument (Malvern Instrument, Worcs, UK).

### Cell culture

Caco-2 cells were obtained from the American Type Culture Collection (Rockville, MD, USA) between passages 25 to 30. Cells were cultured in DMEM medium for 14 d, 10% fetal bovine serum (FBS), 4.5 g/L glucose, 2 mmol/L L-glutamine, 100 U/mL penicillin and 100 U/mL streptomycin; the medium was replaced every two day. Caco-2 cells were seeded in flasks and incubated at 37 °C, under a 5% CO_2_ in air atmosphere. After one week, the cells were harvested and reseeded in 12-well transwell plates at a density of 5×10_4_ cells/cm^2^, and incubated to reach 100% confluence at 37°C, under a 5% CO_2_ in air atmosphere. Transepithelial electrical resistance TEER) was measured to check the integrity of Caco-2 cell monolayers. The transwells consisted of the apical chamber (which simulated the intestinal lumen) and the basal chamber (which would collect the bioavailable iron), which were separated by a polycarbonate membrane with 0.4 µm pores.

### Iron transport in Caco-2 cells

The apical and basolateral surfaces of Caco-2 cells in the transwell were rinsed three times with D-Hanks buffer solution. Then, 500 µl of MEM medium (contained no added iron), previously warmed at 37°C, was added to the basolateral side. Iron transport was started by the addition of 300 µl (1, 10, 20 and 50 µmol/L iron, containing predetermine amount of phytic acid or ZnCl^2^ ) of iron liposomes or ferrous glycinate in D-Hanks buffer solution in the apical side of the transwell. Then, they were incubated for 120 min at 37°C, under 5% CO_2_. After incubation, the transport was stopped by washing the inserts three times with ice-cold 1 mmol/L EDTA in PBS buffer (0.01 mol/L, pH7.4). The basolateral side solution was recovered for the determination of the iron transported across Caco-2 cells.

### Determination of iron

The iron concentration was measured according to the procedure described by Jorhem^20^. Briefly, aliquots of the samples were disrupted to remove organic compounds and to release elemental iron thoroughly by ashing for 10 h at 450°C in a muffle furnace. The inorganic residues were dissolved in 1 mol/L HCl. The determination of iron was performed by graphite furnace atomic absorption spectrophotometry (AAS, Zeenit 700P, Analytik Jena AG, Germany) at 248.3 nm. The calibration curves were performed with standard solutions containing 0-6 µg/mL of ferric chloride in 1% (V/V) nitric acid.

### Data analysis and statistical evaluation

The iron transport was calculated based on the iron amount as determined by AAS and the surface area of the cell monolayer. The iron transport was calculated using the following equation:

Iron transport (pmol/cm^2^)= MA_Fe_/A

where MA_Fe_ is the amount of substance of iron in the basolateral side; A is the surface area of the cell monolayer. Statistical analysis of the data was performed using Matlab software (Version 7.11.0.584(R2010b), MathWorks, Inc. Natick, Massachusetts, USA). Differences in the amount of iron transport were compared using two-way ANOVA. The differences were considered significantly different if P values were less than or equal to 0.05. Variance within treatment groups is expressed as standard deviation (SD).

## Results and discussion

### Effect of incubation time on iron transport from ferrous glycinate liposomes

**Figure 1:**
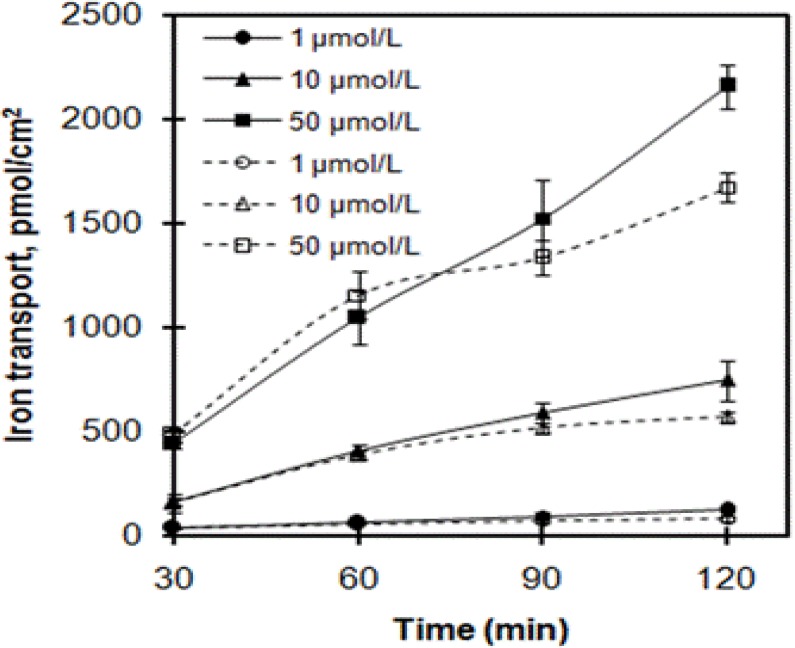
Effects of incubation time on iron transport across Caco-2 cells. Ferrous glycinate liposomes and ferrous glycinate are expressed in solid lineseries and dashed line series, respectively. Values are means ± SD, n = 3

Iron transport across Caco-2 cells treated with increasing incubation time at four levels, 30, 60, 90, and 120 min, are shown in Figure 1. Iron transport of ferrous glycinate liposomes was in agreement with that of ferrous glycinate, which was time-dependent for the period of 120 min, and the iron tended to increase with increasing incubation time. Moreover, iron transport was increased with increasing of iron concentrations. The iron transport from ferrous glycinate liposomes was significantly higher than that from ferrous glycinate (two-way ANOVA; incubation time and iron sources, F[1, 16]=9.38, p=0.0074). Compared to ferrous glycinate, the relative iron transport of ferrous glycinate liposomes at 1, 10, and 50 µmol/L and incubation for 120 min were 146.1%, 131.1%, and 128.9%, respectively, which indicated that the iron of ferrous glycinate liposomes across Caco-2 cells could be more efficient.

Ferrous glycinate liposomes demonstrated higher iron transport than that from ferrous glycinate at the same iron level. The difference in the values of iron transport between the two iron sources was not so apparently in the initial phase or at low iron concentration, whereas the iron transport from ferrous glycinate liposomes was obvious higher than that from ferrous glycinate at the ultimate stage and at high concentration. The higher iron transport of ferrous glycinate liposomes could result from the protection of ferrous glycinate by liposomal vesicles^18^. The lipophile of liposomes was higher than that of ferrous glycinate, so the affinity between ferrous glycinate and cells could be enhanced by the iron encapsulated in liposomes^21^,^22^. In addition, particle sizes of ferrous glycinate liposomes were nano-scale, so the liposomes could behave in nanoparticle effects^23^.

### Effects of phytic acid on iron transport from ferrous glycinate liposomes

**Figure 2:**
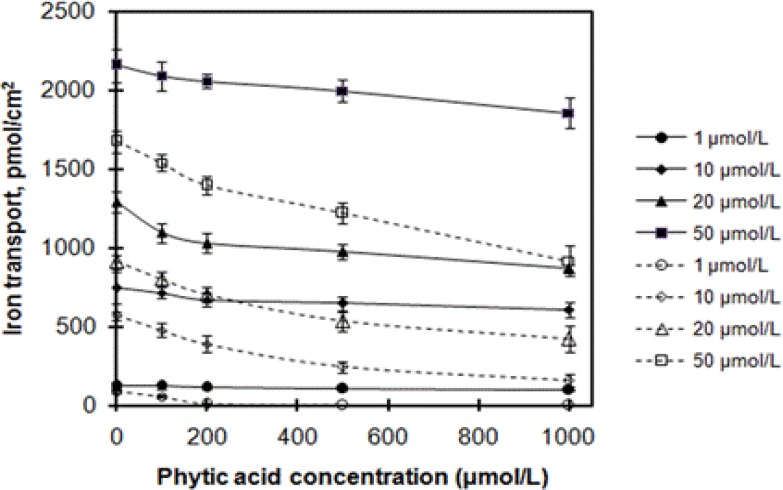
Effects of phytic acid on iron transport across Caco-2 cells. Ferrous glycinate liposomes and ferrous glycinate are expressed in solid line series and dashed line series, respectively. Values are means ± SD, n = 3.

Iron bioavailability was decreased by many inhibitors in the daily diet. Phytic acid extensively exists in vegetable food, such as cereals, beans, and it is a strong inhibitor of non-heme iron transport^24^,^25^. Figure 2 documented the iron transport in response to increasing concentrations of phytic acid. In the presence of phytic acid, iron transport from ferrous glycinate liposomes and ferrous glycinate was evidently inhibited, and iron transport decreased with increasing of phytic acid concentration. However, two-way ANOVA results (phytic acid concentration and iron sources, F[1, 20]=575.15, p<0.001) showed that effects of phytic acid on iron transport from ferrous glycinate liposomes and ferrous glycinate were significantly different. Compared with ferrous glycinate, iron transport from ferrous glycinate liposomes was less inhibited by phytic acid. For example, at the iron concentration of 50 µmol/L, the iron transport at phytic acid concentration of 100, 200, 500, and 1000 µmol/L were decreased by 3.0%, 4.6%, 7.4%, and 14.0% for ferrous glycinate liposomes and by 8.0%, 16.5%, 27.0%, and 45.2% for ferrous glycinate, respectively. Compared to ferrous glycinate, the relative iron transport of ferrous glycinate liposomes at 1, 10, 20, and 50 µmol/L at phytic acid concentration of 100 µmol/L were 213.7%, 149.4%, 136.2%, and 135.9%, respectively.

Phytic acid has proved to be a stubborn inhibitor of iron absorption in humans, which widely exists in cereals and soybeans^11^,^26^. The compound is prone to chelate divalent metal ions and to form insoluble and indigestible chelate complex, which would result in the loss of iron absorption^27^. The results of Figure 2 exhibited that iron transport from ferrous glycinate was seriously decreased by phytic acid. However, iron transport from ferrous glycinate liposomes was less inhibited by the organic acid. The higher iron transport from ferrous glycinate liposomes may be due to the encapsulation of ferrous glycinate in liposomes, and the iron was protected from phytic acid by the phospholipids bilayer membrane, which prevented the formation of insoluble phytate-iron complex^28^.

### Effects of Zinc ion on iron transport from ferrous glycinate liposomes

**Figure 3:**
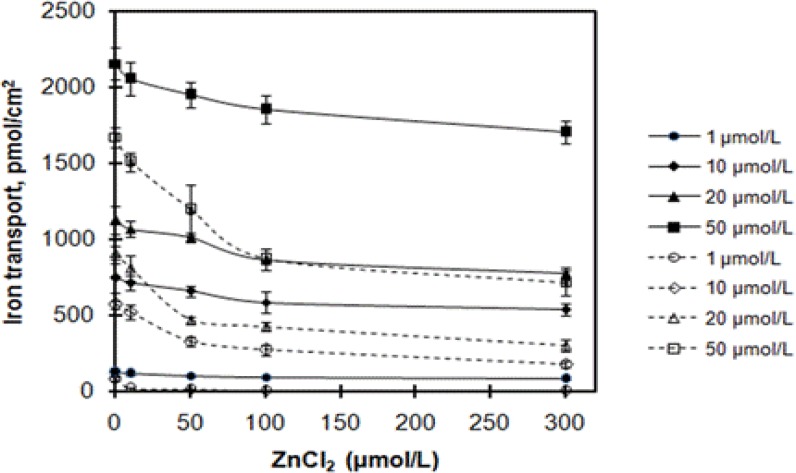
Effects of ZnCl_2_ on iron transport across Caco-2 cells. Ferrous glycinate liposomes and ferrous glycinate are expressed in solid line series and dashed line series, respectively. Values are means ± SD, n = 3.

The interactions of iron and zinc in foods represent critical nutrition issues. The effects of zinc on iron transport are summarized in Figure 3. The addition of zinc at concentrations of 0, 10, 50, 100, and 300 µmol/L decreased iron transport, which was decreased with increasing of zinc concentration. However, judging from the two-way ANOVA results (ZnC_l2_ concentration and iron sources, F[1, 20]=448.64, p<0.001), there were significantly different effects of ZnC_l2_ on iron transport from ferrous glycinate liposomes and ferrous glycinate. Compared with ferrous glycinate, iron transport from ferrous glycinate liposomes was less affected by zinc. For instance, at the iron concentration of 50 µmol/L, iron transport at zinc concentration of 10, 50, 100 and 300 µmol/L were decreased by 4.7%, 9.6%, 14.1%, and 20.9% for ferrous glycinate liposomes and by 9.7%, 28.7%, 48.0%, and 57.2% for ferrous glycinate, respectively. Compared to ferrous glycinate, the relative iron transport of ferrous glycinate liposomes at 1, 10, 20, and 50 µmol/L at the zinc concentration of 50 µmol/L were about 10.3-fold, 2.0-fold, 2.2-fold, and 1.6-fold, respectively. The results implied that liposomes could improve the anti-inhibition of iron. Plenty of literature evidenced that the interaction of iron and zinc in foods was antagonistic effect, which would cause the reduction in bioavailability of the two mineral elements^29^,^30^. Espinoza et al. and Iyengar et al. reported that divalent metal transporter 1 (DMT1) and/or Zip14 (Zrt- and Irt-like protein 14) were the probable iron and zinc interaction site^31^,^32^. Representative results from Figure 3 indicated that iron transport from ferrous glycinate was sharply decreased with increasing of zinc concentration. It could be inferred that the antagonism between iron and zinc has not been eliminated. Compared to ferrous glycinate, the higher iron transport from ferrous glycinate liposomes might be attributed to the fact that the iron was encapsulated in liposomal vesicles. Liposomes could be delivered via membrane fusion, diffusion or phagocytosis^33^,^34^, so iron was more efficiently transported. It could be speculated that ferrous glycinate loaded in phospholipid vesicles could be transported by liposomes rather than DMT1 protein. It was conceivable that ferrous glycinate liposomes could be absorbed through endocytic pathway, and the antagonistic effect between iron and zinc was abated. Pereira et al. affirmed that iron encapsulated in nanoparticles was utilized following acquisition by endocytic uptake^35^. Niu et al. reported that liposomes were likely to be absorbed intact via the M-cell or the epithelia pathways^13^. However, the bilayer membrane of liposomes could be destroyed by divalent metal cation-induced destabilization^36^. And it could lead to leakage of iron from ferrous glycinate liposomes, and the iron transport decreased.

### Effect of particle size on iron transport from ferrous glycinate liposomes

**Figure 4:**
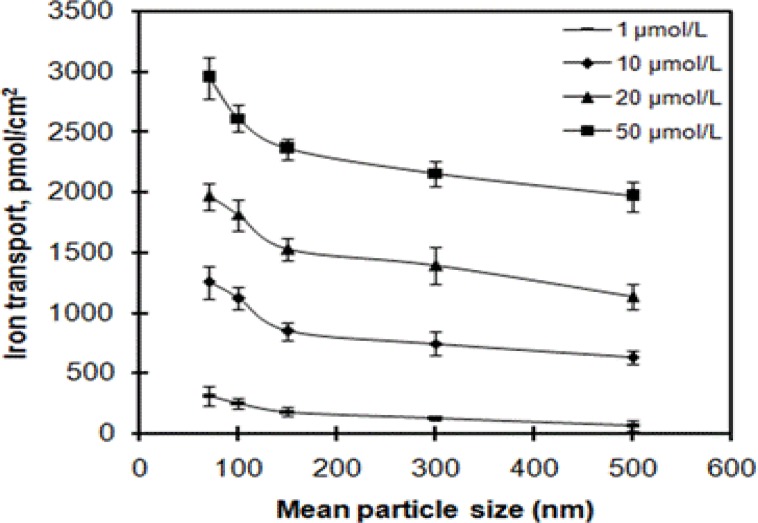
Effects of mean particle size on iron transport across Caco-2 cells. Values are means ± SD, n = 3.

Iron transport of ferrous glycinate liposomes at five particle sizes, 70, 100, 150, 300, and 500 nm were assessed (Figure 4). As shown in Figure 4, iron transport was decreased with particle size increasing of ferrous glycinate liposome at selected iron concentrations. A two-way ANOVA was performed to determine the statistical significance of particle size and iron concentration as factors. Significant effects of particle size and the interaction between particle size and iron concentration interactions were observed ( one-way ANOVA, particle size, F[4, 40]=84.94, p<0.001; two-way ANOVA, particle size and iron concentration, F[12, 40]=4.81, p<0.001). liposomes with smaller particle size of about 70 nm exhibited evidently higher iron transport than liposomes with bigger particle size of 300 and 500 nm (two-way ANOVA; p<0.001 in both cases). Liposomes with particle size of 300 and 500 nm exhibited lower iron transport. The iron transport was not significantly different (p>0.05) between these two particle sizes. An example of the effects of particle sizes was the decrease of the iron transport with particle size increasing at the iron concentration of 50 µmol/L (ANOVA; F[4, 10]=29.91; p<0.001), and compared to that of 70 nm the iron transport was decreased by 11.4%, 20.1%, 26.9%, 33.3% for ferrous glycinate liposomes with particle sizes of 100, 150, 300, and 500 nm, respectively. The obvious size-dependent behavior indicated some kind of size-dependent recognition and internalization of ferrous glycinate liposomes.

It has been revealed that particle size plays a vital role in nanoparticle adhesion to and interaction with biological cells^37^,^38^. Iron transport from ferrous glycinate liposomes was size-dependent, i.e. it increased with the decreasing of particle sizes (Figure 4). In the present study, these results were coincident with previous results in which core material bioavailability from liposomes increased with particle size decreasing^37^,^39^. It was generally supposed that particles within the 100–200 nm size range could be internalized via the pathway of receptor-mediate endocytosis, while particles with larger sizes would be captured by phagocytosis^37^. However, particles in the size range of approximately 2–100 nm could play an active role in mediating biological effects more than being carriers. Signaling processes essential for basic cell functions could be altered by the smaller particles^39^. The exact absorption mechanisms of particles with different sizes need to be elucidated in further studies.

The iron transport offered as ferrous glycinate liposomes was evaluated using Caco-2 cells, as a model of human intestinal epithelia. The results from this study strongly suggest that iron from ferrous glycinate liposomes was transported more effectively than that from ferrous glycinate. The effects of some common inhibitors, such as phytic acid and zinc on, on iron transport from liposomes were obviously decreased, compared to ferrous glycinate. It may be ascribed to the protection of phospholipide bilayer membrane on ferrous glycinate from common inhibitors. The fact quite possibly meant that the transport pathway of iron encapsulated in liposomes was different from that of free-iron. Furthermore, the iron transport was regulated by the size of liposomes, and it decreased with particle size increasing. Liposomes with the sizes of less than 100 nm, among 100–200 nm, and larger than 200 nm could be taken up via different pathways, such as altering signaling processes essential for basic cell functions, receptor-mediate endocytosis, phagocytosis, rather than traditional absorption pathway.

## Conclusion

Liposomes could behave as more than a simple carrier, and iron transport from liposomes could be implemented via a mechanism different from the regulated non-heme iron pathway.
